# Antipsychotics for elderly with psychosis: Deprescribe or continue?

**DOI:** 10.1192/j.eurpsy.2021.185

**Published:** 2021-08-13

**Authors:** P. Mohr

**Affiliations:** 1 Psychiatric Clinic, 3rd Faculty of Medicine, Charles University, Praha, Czech Republic; 2 Clinical Dept., National Institute of Mental Health, Klecany, Klecany, Czech Republic

**Keywords:** Antipsychotics, schizophrénia, drug discontinuation

## Abstract

Maintenance treatment with antipsychotics remains the key principle in the long-term management of psychotic disorders. For some patients, it means life-long use of medication. Continuous drug administration helps to prevent relapses, maintain remission, and achieve functional recovery. Moreover, epidemiological data suggest that antipsychotic treatment significantly reduces mortality rates of schizophrenia patients. On the other hand, some authors argue that antipsychotic drugs may lose its efficacy over time, their long-term exposure results in more harm than benefit. Especially elderly patients are more sensitive to side effects. Several studies which followed-up patient cohorts over the span of several decades found that there are schizophrenia patients who can achieve good functional outcome and full recovery without antipsychotic treatment. Therefore, it is paramount to identify those individuals, particularly among elderly psychotic patients, who can thrive and benefit from timely antipsychotic discontinuation.

**Disclosure:**

No significant relationships.

